# Principles of Disinfectant Use and Safety Operation in Medical Facilities During Coronavirus Disease 2019 (COVID-19) Outbreak

**DOI:** 10.1007/s42399-020-00413-x

**Published:** 2020-07-18

**Authors:** Gen Takagi, Kazuyoshi Yagishita

**Affiliations:** 1 Department of Cardiovascular Medicine, Nippon Medical School, 1-1-5 Sendagi, Bunkyo-ku, Tokyo, 113-8603 Japan; 2 Clinical Center for Sports Medicine and Sports Dentistry, Tokyo Medical and Dental University, Yushima, Bunkyo-ku, Tokyo, 113-8519 Japan

**Keywords:** COVID-19, Hemodialysis, Intensive care unit, Hyperbaric oxygen therapy, Disinfection

## Abstract

Medical collapse became a major concern under coronavirus disease 2019 (COVID-19) outbreak; prevention of medical accidents is essential during disinfection either. The objective of this review is to enhance the awareness regarding the safety aspects towards infection prevention practices and to offer solutions for safe patient care practices including side effects of disinfectants and precaution in specific medical facilities especially in hemodialysis rooms, intensive care unit, hyperbaric oxygen therapy (HBO) chambers, or patient transport vehicle. Literature was researched that was obtained from studies of human coronavirus infections, including the severe acute respiratory syndrome (SARS) and the Middle East respiratory syndrome (MERS), and created a summary of the characteristics of these disinfectants. This review is not intended to replace infection prevention policies and procedures established by hospitals, and manufacturers, but to provide some update confidence in the safety measures that each medical facility already uses and to offer additional input that should optimally reduce the risk of infection.

## Introduction

**Fig. 1:**
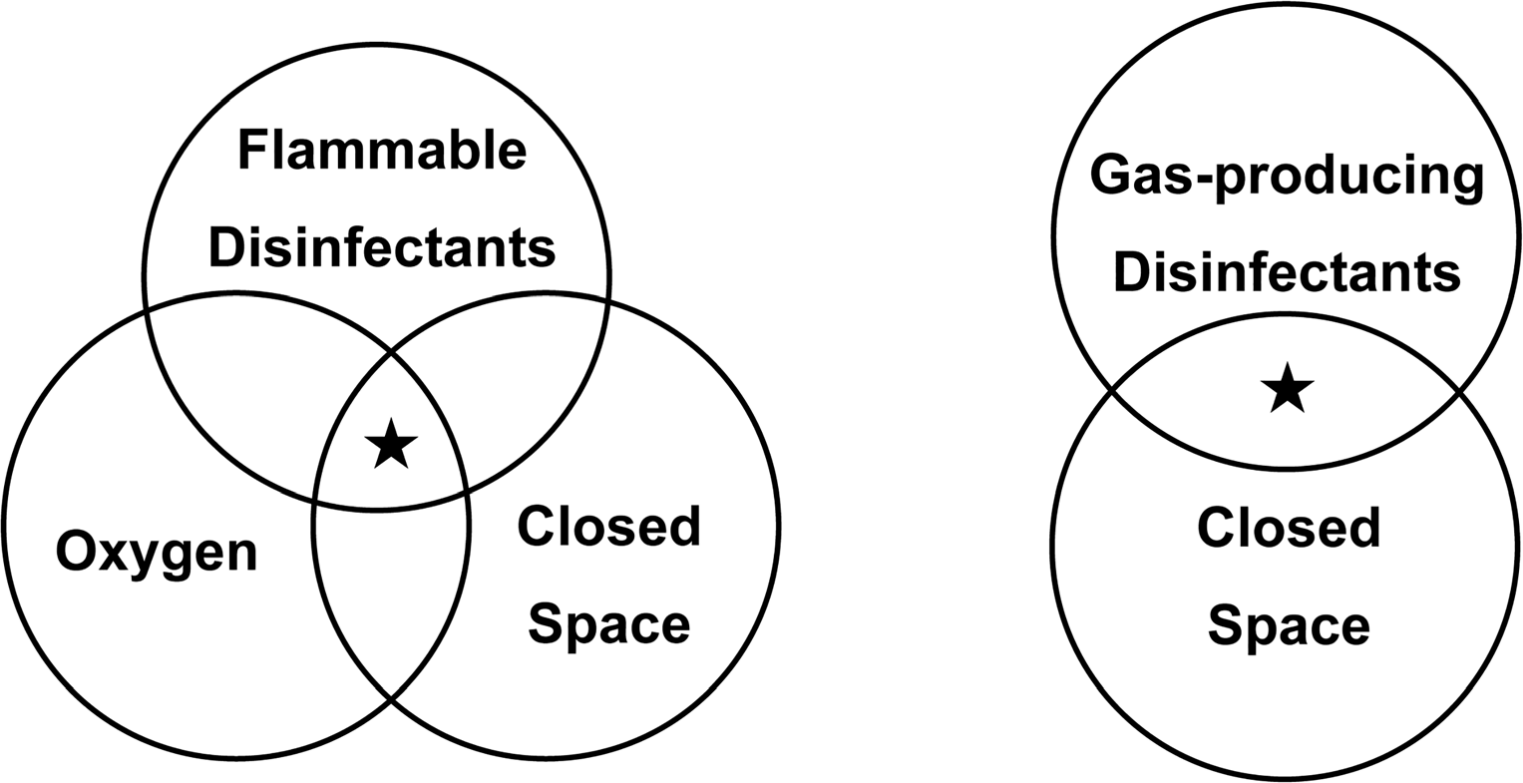
Conceptual diagram of risk overlap

The novel coronavirus disease 2019 (COVID-19) is caused by severe acute respiratory syndrome coronavirus 2 (SARS-CoV-2). As human coronaviruses can remain infectious on inanimate surfaces for several days [1], surface disinfection with chemicals is inevitable. Coronavirus is an RNA enveloped virus [2] and generally not highly resistant to disinfectants and can also be inactivated by heat and ultraviolet (UV) irradiation [3]. However, inadequate use of disinfectants causes side effects to the medical staff, and inadequate use in poor ventilation may result in fire, gas poisoning, explosion, or equipment corrosion. Disinfection in a non-critical or semi-critical area that does not have negative pressure rooms deserves attention. As illustrated in Fig. 1, a special attention must be paid to situations where the risk factors overlap. Flammable disinfectants under oxygen use may cause an explosion triggered by static electricity or excessive heat; also, poisoning may occur with gas producible disinfectants under poor ventilation. Thus, this review focuses on the infection prevention practices and safety aspects including equipment protection measures against potential damage. The aim of this review is to update the confidence for all the caregivers who already complying with health and safety recommendations and following strict training. The efficacy data was obtained from studies of two types of human coronavirus infections, the severe acute respiratory syndrome (SARS) and the Middle East respiratory syndrome (MERS), and created a summary of the characteristics of these disinfectants.

## Alert for Contamination Prevention

1. Limit the access of visitors to closed care spaces such as intensive care unit, hemodialysis rooms, and hyperbaric oxygen therapy chambers, because any individual may have a chance of COVID-19 infection. Also, standard precautions must be performed [4].
2. Any treatments should be performed in well-ventilated spaces upon identification of COVID-19 infection, unless their benefits outweigh the infection risk, for example, when a treatment is lifesaving or is required as part of intensive care such as patient transport vehicle [5]. Transmission-based precautions is necessary for this condition [4].
3. Avoid encounter of outpatients and inpatients at the same place. If multiple patients need to be treated at the same space at the same time, limit the number of patients and keep the patients as far as possible from each other (ideally 1 m or more) unless partition was prepared.
4. There is ability for SARS-CoV-2 to remain in the context of remarkably high bioburdens such as body fluid, vomit, and stool. Wear gloves when processing [4].
5. There is a report that 30% of medical staff was infected in a hospital; a safety measure for medical staff is also important [6].

## Alert for Procedures

1. General cleaning must follow the Centers for Disease Control and Prevention (CDC) guidance for standard, contact, and airborne precautions, including the use of eye protection and personal protective equipment (PPE) [4]. When reusing N95 mask, proper management such as disinfection measures is important [7].
2. If a dedicated isolate procedure is not available in order to share a single room (hemodialysis room or HBO chamber etc.), schedule treatments for inpatients first (in the morning), and for outpatients after (in the afternoon), such those patients are isolated from each other. Ensure that disinfect cleaning is performed during and after each procedure.
3. When not in use, such as at night, and unless there is an emergency, open the treatment space and ventilate it.

## Alert for Disinfection

When disinfecting a SARS-CoV-2-contaminated space or surface, exposure time and concentration of disinfectants are important. Follow the recommended disinfectants and concentration as indicated by the CDC and ensure the environmental disinfection procedure based on your local decontamination guidelines. In addition, please do not forget the caregiver’s safety as well [4]. Many disinfectants cause side effects such as eye irritation, mucous irritation, and contact dermatitis. Thus, environmental disinfection needs to be performed with adequate personal protective equipment (PPE) and training. Also, some disinfectants have a chance of explosion and gas poisoning with their flammability and toxicity. Even if the chance is rare, the accident level is high. Thus, check for a room- or area-specific conditions associated with the use of the disinfectant, such as flammability (especially under oxygen use), and check for a risk of toxic gas production during the disinfecting process. Do not be sprayed in closed spaces. Confirm with the vendor that the disinfectant is safe to use on the specific equipment materials and will not cause corrosion or breakdown. To understand the advantages and disadvantages of each disinfectant, please refer to our quick guide for disinfectant use (Table 1).

**Table 1:** Quick guide for disinfectant use

Chemical name	Bacteria	Envelope virus	Flammable or toxic gas	Human		Instrument	
				Hand	Wound	Metal or glass	Acrylic resin
UV-C	●	●	-	✖	✖	M	●
Ethanol	●	●	F	●	✖	●	✖
Hydrogen peroxide	●	●	F	●	●	●	●
Phenolic cresol	●	●	F, G	✖	✖	✖	✖
Glutaraldehyde	●	●	G	✖	✖	●	●
Sodium hypochlorite	●	●	G^1^	✖	✖	✖	✖
Formaldehyde	●	●	G	✖	✖	●	●
Benzalkonium chloride	●	W	-	●	●	●	●

A filled circle indicates effective or recommend. An X indicates ineffective or not recommended. *UV*, ultraviolet; *M*, practicable for metal but not for glassware; *F*, flammable; *G*, toxic gas-producing; *W*, weak

^1^When mixed with ammonia

### Flammable Agents

1. Ethanol (70–95%) or isopropanol (50–100%) inactivates SARS-CoV-2 [8]; however, it must be used with adequate ventilation. Wipe with an impregnated sheet followed by forced ventilation and allow drying time at least 30 min. These substances are volatile and flammable and can cause an explosion when used in closed spaces that contains higher oxygen concentration. Ethanol or isopropanol may cause acrylic resin degradation when disinfecting synthetic resin materials.
2. Hydrogen peroxide, 0.5%, inactivates some coronaviruses, including SARS-CoV-2 [1]; however, it is explosive with heat.
3. 0.5% of phenolic cresol must be used with adequate ventilation. This compound is flammable and is toxic by inhalation. Rubber, aluminum compounds, zinc, lead, plated iron, and polyethylene can be corroded by this compound [9].

### Gas Produce or Toxic Agents

1. Avoid using glutaraldehyde in closed spaces. It may cause mucous membrane irritation in the eyes and in the respiratory system, as well as skin side effects [10]. Use glutaraldehyde only in ventilated areas, according to each medical institution’s guidance.
2. Avoid using formaldehyde in closed spaces. It may cause mucous membrane irritation in the eyes and in the respiratory system, as well as skin side effects.

### Corrosion or Breakdown

1. Avoid using sodium hypochlorite (effective concentration, 0.21%) on metals, on acrylic resins either, because it is highly alkaline and has a strong corroding effect [11].
2. Avoid using potassium peroxymonosulfate with spray, because it is toxic by inhalation. Aluminum compounds, zinc, and plated iron can be corroded by this compound.
3. The disinfecting effect of chlorhexidine gluconate on RNA viruses has not been proven [1]. Also, the metal corroding effect was reported [11].

## Recommended Disinfectants for Equipment in Closed Spaces

1. 200–280 nm (UV-C) light can quickly disinfect the air [12], inactivating SARS-CoV-2 [8]; however, the shadow of the irradiation is not disinfected. Avoid direct irradiation to organic glassware such as polyvinyl chloride. It is possible that UV-C is safe for skin genotoxicity [13]; however, its effect to the naked eye that may cause impaired vision is not confirmed [14]. Also, UV light does not penetrate plastic and glass [8].

## Disinfectants that Are Not Suitable Against SARS-CoV-2

1. The disinfecting effect of quaternary ammonium salts (benzalkonium chloride, benzethonium chloride) on RNA viruses such as coronaviruses is weak [1].
2. The disinfecting effect of alkyl diaminoethyl glycine hydrochloride on RNA viruses has not been proven.
3. Other disinfectants approved by the United States Environmental Protection Agency (EPA) are listed on the agency’s website [15]

## Conclusions

An accurate understanding of the advantages and disadvantages of disinfectants and operation may provide updated confidence in safety measures and may reduce the risk of infection.
